# Defining the Criteria for Selecting the Right Extended Reality Systems in Healthcare Using Fuzzy Analytic Network Process

**DOI:** 10.3390/s25103133

**Published:** 2025-05-15

**Authors:** Ali Kamali Mohammadzadeh, Maryam Eghbalizarch, Roohollah Jahanmahin, Sara Masoud

**Affiliations:** 1 Department of Industrial and Systems Engineering, Wayne State University, Detroit, MI 48201, USA; alikamali@wayne.edu (A.K.M.);; 2 Department of Health Services Research, Division of Cancer Prevention & Population Sciences, The University of Texas MD Anderson Cancer Center, Huston, TX 77030, USA; meghbalizarch@mdanderson.org

**Keywords:** extended reality, analytic network process, fuzzy theory, healthcare

## Abstract

**Highlights:**

**What are the main findings?**

- The first comprehensive Fuzzy Analytic Network Process (FANP)-based decision support system (DSS) tailored to extended reality (XR) systems in healthcare is proposed to systematically evaluate XR technologies for healthcare applications by integrating the Supply Chain Operations Reference (SCOR) model and FANP.
- A structured analysis of XR systems reveals that reliability and responsiveness are the most critical factors, with safety, accuracy, and user control being top-ranked sub-factors.

**What is the implication of the main finding?**

- The proposed model enables data-driven and expert-informed selection of XR systems, improving alignment with healthcare performance needs.
- Insights from sensor-based evaluation and weighted criteria support XR system design, procurement, and strategic implementation in clinical settings.

**Abstract:**

In the past decade, extended reality (XR) has been introduced into healthcare due to several potential benefits, such as scalability and cost savings. As there is no comprehensive study covering all the factors influencing the selection of an XR system in the healthcare and medical domain, a Decision Support System is proposed in this paper to identify and rank factors impacting the performance of XR in this domain from an engineering design perspective. The proposed system is built upon the Supply Chain Operations Reference (SCOR) model supported by a literature survey and experts’ knowledge to extract and identify important factors. Subsequently, the factors are categorized into distinct categories, and their relative importance is specified by Analytic Network Process (ANP) models under a fuzzy environment. Two fuzzy approaches for the ANP models are compared, and the results are analyzed using statistical testing. The computational results show that the ranking agreement between the two fuzzy approaches is strong and corresponds to the fact that both approaches yield the same ranking of primary factors, highlighting the significance of reliability as the topmost factor, followed by responsiveness, cost, and agility. It is shown that while the top three important sub-factors are identical between the two approaches, their relative order is slightly varied. Safety is considered to be the most critical aspect within the reliability category in both approaches, but there are discrepancies in the rankings of accuracy and user control and freedom. Both approaches also consider warranty and depreciation costs as the least significant criteria.

## 1. Introduction

Extended reality (XR) is a term used to describe all immersive technologies that exist within the reality–virtuality continuum. The reality–virtuality Continuum is a theoretical framework that describes the relationship between real and virtual environments [1]. The continuum ranges from the real environment at one end to the virtual environment at the other end, with augmented reality (AR) and mixed reality (MR) in between. The reality–virtuality continuum helps to categorize and compare different types of immersive technologies based on their level of immersion and interaction with the physical world. It also provides a framework for designing and developing immersive experiences that fit within a particular point on the continuum. Virtual reality (VR) creates a completely digital environment that simulates the physical world or an imaginary one. AR overlays digital content on top of the real world, while MR blends digital objects with the physical world in real time.

Within recent decades, XR has been gaining increasing interest across different fields, including retail [2], education [3], manufacturing [4], and healthcare [5]. Technological advancements have transformed healthcare by introducing innovative approaches that enhance patient care and clinical outcomes, with XR technologies gaining increasing recognition for their potential across both clinical practice and research settings [6]. XR has made its debut in the healthcare sector, starting with surgical procedures, and has since expanded to medical training, patient care, and various other areas [5]. Recent evidence indicates that XR is of benefit for patient education, educating and training medical students, surgical planning, medical imaging, rehabilitation, and pain management [7]. Additionally, it has been incorporated into the treatment of several medical conditions like neurodegenerative disorders (e.g., post-traumatic stress disorder (PTSD), panic disorders, special phobias, autism spectrum disorder (ASD)), dental medicine, orthopedics, obesity, and so forth [5,8].

XR can offer an engaging and authentic user experience that is practical for teaching and providing immediate guidance during complex medical emergencies, diagnostic procedures, interventions, and procedural applications [9]. Moreover, for beginners, XR can provide extra support to enhance their proficiency in making medical decisions, utilizing medical devices and equipment, and executing medical procedures. XR enables healthcare professionals to access people, information, and experiences that were once inaccessible, thereby reducing the geographical barriers between the delivery of value-based healthcare and patients [9]. The most noteworthy benefit of XR in the healthcare industry could be its ability to provide communal and shared experiences. In the past, medical professionals might not have been able to empathize with their patients’ medical issues since they were not sick themselves. However, with the advent of XR, this could change, as medical personnel can now experience how it feels to be unwell and gain a better understanding of their patients’ perspectives. XR involves the use of a specialized headset to simulate an immersive 3D virtual environment, enabling users to feel physically present in a synthetic space. Through interactions with virtual characters (avatars) and real-time feedback using audio, visual, and haptic stimuli, medical personnel can engage in simulations that replicate patients’ experiences, thereby fostering empathy and improving patient care [10].

Recent advances in sensor technologies have played a pivotal role in shaping the development and application of XR systems. Sensors such as inertial measurement units (IMUs), depth cameras, RGB-D sensors, LiDAR, eye trackers, and physiological sensors are now integral to most XR platforms. These sensing components enable spatial tracking, environmental awareness, and user interaction, forming the technical foundation for immersive experiences. The richness and reliability of sensor data directly affect system responsiveness, safety, and realism—factors that are especially critical in high-stakes environments such as healthcare [11].

The type and configuration of sensors differ significantly across VR, AR, and MR systems. VR systems often emphasize motion tracking and visual immersion, leveraging IMUs and optical tracking to replicate physical motion within digital spaces. AR systems require more advanced sensing, such as simultaneous localization and mapping (SLAM) and depth estimation, to anchor virtual content to the physical world accurately. MR systems go a step further by integrating multimodal inputs such as eye gaze, gesture recognition, and contextual awareness to allow real-time dynamic interaction between digital and physical elements. These differences in sensing complexity not only influence the cost and form factor of XR systems but also determine their suitability for specific clinical and operational tasks. For instance, MR systems like HoloLens 2 are better suited for image-guided surgery, while simpler VR systems may suffice for immersive procedural training.

The evaluation of XR systems is far from a simple, straightforward task, given that numerous heterogeneous factors should be considered in an integrated manner. Recent work highlights the potential of integrating XR in healthcare and notes challenges in system-level evaluation and integration [5], although formal qualitative and quantitative comparison frameworks remain underdeveloped. This paper introduces an effective Decision Support System (DSS) tailored for the evaluation and selection of XR systems in the healthcare sector. Our DSS is founded on a framework that integrates the Supply Chain Operations Reference (SCOR) with the Fuzzy Analytical Network Process (FANP) model. The presented system aligns with the hallmark characteristics of a DSS by integrating diverse data sources, utilizing a hierarchical model, and employing sophisticated algorithms like FANP to facilitate informed decision-making. This combination allows for systematic identification and prioritization of the influential criteria essential for assessing XR systems in healthcare settings. Additionally, two state-of-the-art fuzzy approaches for the ANP models are compared, and the results are analyzed using statistical testing. The proposed SCOR-FANP system allows decision-makers to evaluate and compare available XR systems in a systematic manner to select the most suitable one. The proposed approach provides a comprehensive evaluation of different factors affecting the performance of XR systems in healthcare. It also allows researchers to have an early evaluation of the XR system during the design phase. Our seminal contribution is rooted in its application to healthcare XR system selection, synergistically combined with the SCOR model. This blend and the consequent insights present a distinctive contribution to the existing literature.

The remainder of this paper is organized as follows. Section 2 reviews the body of literature and highlights the most important features of XR systems. Section 3 describes the methodology of the study. Section 4 discusses the experimental results. Section 5 presents a performance evaluation of existing XR technologies based on the proposed criteria. Finally, Section 6 concludes the paper.

## 2. Literature Survey

This section provides a comprehensive review of the literature on the applications of XR technologies in healthcare. We categorize the literature into distinct sections, focusing on clinical applications, technological advancements, and evaluation studies.

**Clinical Application-Based Studies.** XR technologies have been brought into healthcare during the past decade due to several potential benefits, such as scalability, increased motivation, and cost savings. The expansion of XR in the healthcare industry has the potential to change how medical services are provided to autistic children, PTSD and depression patients, patients undergoing surgeries, and patients with brain injuries, to name a few [5]. Among the medical areas in which XR technologies are being used, medical education and training, surgical simulation, medical diagnostics, neurological rehabilitation, psychotherapy, and telemedicine can be mentioned [12]. VR has been used therapeutically to treat posttraumatic stress disorder, anxiety disorders, phobias, schizophrenia, addiction, eating disorders, and obesity [13,14,15,16,17]. Patients are more at ease dealing with challenging situations “virtually” rather than in real life since they are aware that the digital environment is not real, and it is likely that learning new behaviors could be applied in the real world [17]. Augmented reality can be helpful in the planning of surgical interventions and in communicating potential medical complications to patients and their families in the context of patient care [18]. Ref. [8] proposed that XR technologies could significantly improve adherence to behavioral interventions and challenged researchers to explore XR-based interventions in treating adolescent obesity. Ref. [14] conducted a review of XR and telehealth interventions for children and adolescents with ASD, aiming to verify their efficacy and validity. Ref. [7] wrote a chapter outlining the established clinical applications of XR, including mental well-being, pain management, physiotherapy, and rehabilitation. Ref. [19] conducted a systematic review investigating the utilization of XR-based therapies for anxiety disorder patients, exploring the perceptions and experiences of patients and healthcare providers, and comparing the effectiveness of different XR approaches in anxiety disorder treatment.

**Technology-Based Studies.** Using whole-slide microscopic images, Google’s AR Microscope employed machine learning to detect cancer in real-time [13]. The proposed system enables integration of artificial intelligence (AI) into routine workflows, which was demonstrated to be useful in the detection of prostate and metastatic breast cancer [14]. A systematic review [20] indicated that integrating AI, XR, and human digital twin-based solutions can reduce technical errors and provide a universal framework for advancing personalized cardiology. Ref. [5] discussed the potential impact of XR in transforming the healthcare industry, outlining its use cases, challenges, XR tools and techniques for intelligent healthcare, recent developments in XR-based healthcare services, and the potential benefits and future aspects of XR in the medical domain. Ref. [21] provided a comprehensive overview of XR’s theoretical potential, highlighting its technical advancements and biomedical applications. Ref. [9] proposed a standardized taxonomy of essential XR capabilities and described important functional characteristics that could serve as a conceptual design and development framework for XR-based medical training and real-time clinical guidance for astronauts during deep space missions.

**Evaluation-Based Studies.** Evaluating the effectiveness of XR systems is still an open question. Among the studies on evaluation and/or comparison of XR systems, most of which focused on qualitative assessment of XR systems [22,23,24,25]. Among other industries other than healthcare, Ref. [26] evaluated the application of AR devices from a process point of view in the manufacturing industry. Ref. [27] critically evaluated the use of AI and XR in real-world biomedical settings, comparing their outcomes to traditional healthcare practices and illustrating their effectiveness through case studies. Ref. [28]’s investigation also examined the integration of AI, XR, and VR in biomedical applications, highlighting their roles in diagnosis, treatment, and medical training. Ref. [29] enhanced the transparency and quality of reporting in early-phase clinical evaluations of XR applications by applying the Delphi method. Ref. [30] identified and highlighted the role of immersive technologies—including VR, AR, MR, XR, the metaverse, and gamification—in supporting healthcare responses to COVID-19. The literature on the subject shows that there is a need for a comprehensive quantitative DSS for evaluating XR systems in healthcare.

To build upon these findings and better understand how XR systems can be optimized for different healthcare applications, it is crucial to examine the role of sensing technologies that underpin XR platforms. The type and configuration of sensors integrated into XR systems directly influence their usability, fidelity, and contextual appropriateness for clinical interventions. Table 1 presents a comparative overview of the primary sensing technologies used in XR systems, highlighting their roles in determining system performance and relevance to healthcare applications. It categorizes sensors such as IMUs, optical trackers, depth cameras, LiDAR, eye trackers, gesture sensors, physiological sensors, and SLAM techniques based on their technical functions, XR domain usage, and clinical applicability. By aligning sensor capabilities with their impact on XR fidelity and medical integration, the table bridges technical specifications with real-world healthcare needs.

### Gap Analysis

While several researchers have explored the potential of XR technologies in the medical field, there is a lack of work proposing a DSS that addresses the criteria influencing the selection/evaluation of XR systems in healthcare. To the best of our knowledge, this study is the first research paper that presents a DSS based on a multiple-criteria analysis for the evaluation and selection of XR systems to be applied in the healthcare sector. The proposed system utilizes a combination of the SCOR model, a literature survey, and expert knowledge to identify and rank influential criteria for evaluating XR systems in healthcare. The ANP model is used to determine the relative importance of each criterion under a fuzzy environment. Fuzzy environments help to deal with the uncertainty and imprecision surrounding the opinion of our experts and literature, allowing for a more flexible and nuanced approach to factor identification and ranking [32,33,34,35]. Two state-of-the-art fuzzy approaches are compared to provide a more comprehensive analysis. The proposed system allows decision-makers to evaluate and compare available XR systems systematically to select the most suitable one while providing researchers with an early evaluation during the design phase. Overall, this approach provides a comprehensive evaluation of different factors affecting the performance of XR systems in healthcare, aiding in effective decision-making.

## 3. Materials and Methods

**Figure 1:**
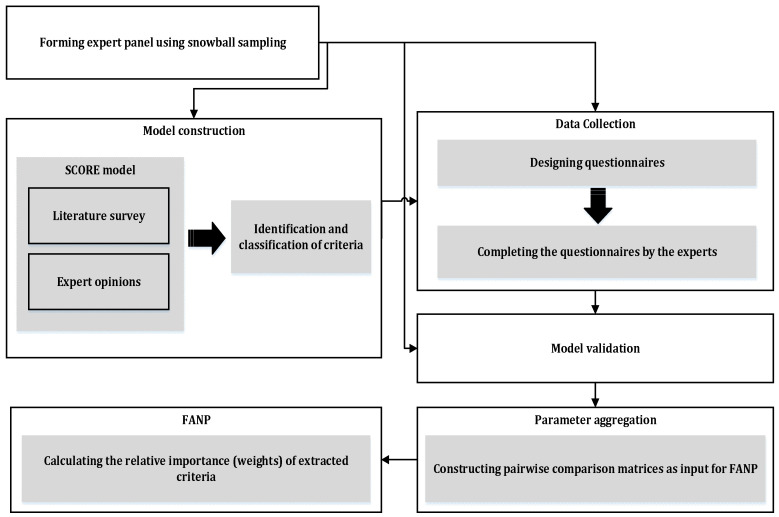
The proposed research methodology.

Our proposed multiple-criteria analysis approach consists of five phases: model construction, data collection, model confirmation/validation, model parameter determination, and calculating local and global weights, as demonstrated in Figure 1.

### 3.1. Model Construction

Based on an extensive review of the literature, the most critical evaluation criteria for XR systems in the healthcare domain are first identified and subsequently classified. Loosely based on the well-established SCOR model [36,37], this paper provides a taxonomy of XR system selection criteria and classifies them into distinct classes.

The SCOR model, with its wide recognition for aiding supply chain strategy formulation, serves as a robust foundation. Its key attributes, like providing a standard description of supply chain processes, performance metrics, and best practices, enable a systematic approach to classify and evaluate XR systems. Leveraging SCOR’s principles allows this paper to develop a coherent taxonomy that is adaptable to the evolving needs of the healthcare industry. It breaks down the criteria for XR system selection in healthcare into four overarching classes (reliability, responsiveness, agility, and cost), further subdivided into 18 specific criteria.

Reliability: The ability to perform tasks as expected. An XR system is expected to be accurate, safe, and convenient.

- Accuracy: The degree to which the result of a measurement or calculation conforms to the correct value or a standard [38]. This definition includes concepts such as precision and recall.
- Safety: The condition or state of being free from harm, injury, or loss [39,40].
- Ease of installation and use: This includes features such as weight, if one or both hands are free during usage, and help and documentation [37].
- User control and freedom: The ability of the user to freely move around and perform intended tasks with full control [41].

Responsiveness: The speed and timeliness of service delivery.

- Feedback: Refers to the response time of the system and its ability to provide real-time feedback [26,42].
- Setup time: The time interval necessary to prepare the system for operation [42,43].
- Maximum usage time: The maximum time that the system can operate without interruption [43].
- Interaction method: Outlines the usability of the specific XR system. It can be vocal, gestural, touching, physiological, or a combination of more than one method [41].
- Information update gap: How fast the XR system allows updating information from/to the central system, thus supporting a more responsive process [42].

Agility: The capacity to effectively sense and respond to external influences/stimuli.

- Supported tech: It can support AR, VR, MR, or a combination of all [26].
- Expandability: The degree to which a system could easily extend or upgrade with new functionalities and abilities to address new requirements [39].
- Operating environment: Refers to the environments (indoor, outdoor, and severe) in which the system can operate [42].
- Operating range: The maximum allowable area where the system can operate [26].

Costs: All the costs associated with operating the processes.

- Purchase cost: The total cost paid for the acquisition of the XR system [44].
- Operating cost: All the required costs related to the operation of the XR system [45].
- Maintenance cost: The sum of the cost of preserving the system [39].
- Warranty cost: The cost paid for obtaining a warranty for the XR system [46].
- Depreciation cost: The reduction in the initial value of the machinery resulting from its exploitation [46].

**Figure 2:**
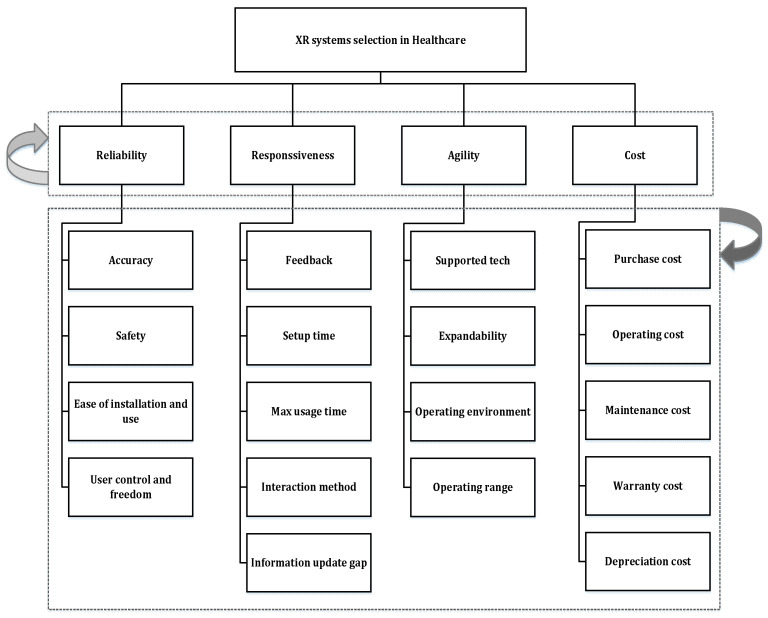
The hierarchical model for evaluating XR systems in healthcare.

Figure 2 graphically showcases the hierarchical model based on this taxonomy.

### 3.2. Data Collection

Employing the snowball sampling method, the XR experts are opted to confirm, identify, and categorize the factors and sub-factors.

#### 3.2.1. Snowball Sampling Method

Snowball sampling is a sampling technique where primary subjects recruit future study subjects among their acquaintances. This technique can be used to identify experts in a certain field. Locating hidden populations and low cost can be mentioned as advantages of the snowball sampling method. Snowball sampling starts with a convenience sample of initial subjects [47]. The initial subjects serve as “seeds”, through which the sample consequently expands through several steps, just like a snowball growing in size as it rolls down a hill [48]. For this work, the opinions of 15 experts in the intersection of XR and healthcare are solicited. All experts met strict eligibility criteria regarding domain experience, graduate qualification, and peer-reviewed contributions. Having a group of five to eight experts suffices for training our proposed model. As an example, Ref. [48] initiated with a snowball sample size of three internet of things experts which later was expanded to a team of eight. We acknowledge that the snowball referral used to contact some participants may have introduced a degree of network-homogeneity bias.

#### 3.2.2. Questionnaire Design

To identify and prioritize the evaluation criteria for XR technologies in healthcare, the FANP—a multi-criteria decision-making method—was employed, as it is well-suited for capturing the complexity and interdependence of evaluation criteria under uncertainty.

The questionnaire was designed based on the validated model proposed by [49], commonly used in prior decision analysis research. The evaluation framework was structured as a network, allowing for the consideration of interdependencies among criteria and sub-criteria. To gather input, we developed a set of pairwise comparison questions. For each pair of criteria, participants were asked to assess their relative importance. Each pairwise comparison asked participants to evaluate the relative importance of one criterion over another. For instance, within the “reliability” cluster, one question was: “How important is Accuracy in comparison to Safety?” This approach was applied consistently across all other criteria and sub-criteria clusters as well.

Respondents expressed their judgment using a linguistic scale (e.g., equally important, moderately more important, etc.) provided in Table 2, which was then converted to a Triangular Fuzzy Number (TFN) to account for the ambiguity and subjectivity of human judgment. This conversion allowed us to represent expert preferences more realistically in the presence of uncertainty. It should be noted that, first, using a pre-approved questionnaire can help validate the questionnaire, and second, homogeneity and clustering as a guarantee for validity insurance are considered [50].

### 3.3. Model Validation

To ensure the robustness and relevance of the proposed model, a comprehensive validation process was undertaken.

#### 3.3.1. Expert-Based Qualitative Validation:

**Preliminary Model Review**: Initially, a preliminary version of the model was presented to a select panel of domain experts. These experts, with substantial experience in both healthcare and XR systems, meticulously reviewed the model’s structure, criteria, and sub-criteria.

**Feedback and Refinement**: Through focused group discussions and one-on-one interactions, the experts provided feedback on the relevance, comprehensiveness, and clarity of the model components. This iterative process allowed for the identification of any overlooked criteria, potential redundancies, or ambiguities.

**Final Model Endorsement**: After incorporating the suggested modifications, the refined model was once again presented to the expert panel for a final review. Their consensus endorsement confirmed the model’s validity and relevance in the target domain.

#### 3.3.2. Questionnaire Distribution:

Once the model’s validity was ascertained through expert endorsement, the next phase was the distribution of the questionnaire.

**Development of the Questionnaire**: The questionnaire was designed to capture the relative importance and interrelationships among the model’s criteria and sub-criteria. It integrated the findings from the literature review with insights from the preliminary expert review.

**Pilot Testing**: Before the full-scale distribution, the questionnaire underwent pilot testing with a subset of experts. This ensured clarity, appropriateness, and the feasibility of the questions. Feedback from this phase further fine-tuned the questionnaire.

**Full-Scale Distribution**: The finalized questionnaire was then disseminated among a broader set of experts in the field. Their responses facilitated the quantitative analysis of the model’s components and their interrelationships. Expert input was gathered through successive rounds over four weeks, with feedback loops until all participants confirmed their final responses. The complete questionnaire is provided in Appendix B.

Through this rigorous validation process, we ensured that our model not only stands up to academic scrutiny but also aligns well with practical, real-world insights and expertise.

### 3.4. Parameter Aggregation

In FANP, the results of pairwise comparison made by the experts are aggregated, and the parameters are calculated as follows: The responses are mapped to TFNs using the scale specified in Table 2. The weights of the experts for each question are calculated using the following equation (weight of expert *j* for question *i*):

(1) $${Weight}_{ij}=\frac{1}{\log\left({distance}_{ij}+1\right)+1},\;$$

where *distance_ij_* is the difference between the response of expert *j* to question *i* and the average of all experts’ responses to question *i*. The aggregated TFN associated with each comparison is calculated based on the weighted average and standard deviation for each question. The aggregated TFNs form the pairwise comparison matrices.

### 3.5. Calculating Criteria Weights

A fuzzy ANP-based model is used to calculate the local and global weights due to its ability to address more generalized relations than AHP. Furthermore, its combination with fuzzy theory is due to the inability of the original ANP model to handle the imprecision and subjectivity in the pairwise comparison process made by the experts. The versatility of ANP and FANP methodologies is evident not just in healthcare but also in diverse applications. For instance, Refs. [52,53,54] are testament to this adaptability and underline the adaptability of these methodologies. From flood vulnerability assessments to soil erosion susceptibility evaluations and beyond, the common thread remains the robustness of ANP and FANP in navigating intricate decision-making processes. Such cross-domain insights accentuate the relevance of ANP and FANP for XR system selection in healthcare [52,53,54].

Let the TFN ($l_{ij},\;m_{ij},\;u_{ij}$) be the elements of the pairwise comparison matrix between criterion i and j (named as pairwise comparison ratios), where $l_{ij},\;m_{ij}$, and $u_{ij}$ indicate the smallest, the most promising, and the largest possible value describing a fuzzy event, respectively [55]. Several approaches have been proposed to extend the ANP model to a fuzzy setting. In this paper, two primary approaches are focused on, which are aimed at determining the final weights of the criteria and prioritizing them accordingly. Table 3 provides a comparative summary of FANP Approaches A and B, outlining their methodological differences, computational structures, advantages, and complexity.

- FANP approach A

This approach, which is based on the model proposed by [49], involves the utilization of fuzzy pairwise comparison judgments instead of exact numerical values for the comparison ratios. By employing this method, the original fuzzy prioritization problem can be transformed into a non-linear program. This is a three-step procedure which is described in detail as follows [56]:

**Step A-1.** Using pairwise comparison matrices, determine the local weights of the factors and sub-factors. Using the following mathematical programming model, the local weights of the factors and sub-factors are calculated:

(2) Maxλ

Subject to: 

(3) $$\left(m_{ij}-l_{ij}\right)\lambda w_{j}-w_{i}+l_{ij}w_{j}\leq 0$$

(4) $$\left(u_{ij}-m_{ij}\right)\lambda w_{j}+w_{i}-u_{ij}w_{j}\leq 0$$

(5) $$\sum_{k=1}^{n}w_{k}=1,w_{k}>0,k=1,2,\ldots ,n$$

(6) i=1,2,…,n−1,j=2,3,…nj>i, 

where w and λ denote the local weight vector and the consistency index. If the optimal objective function of the programming model is positive, it means that the set of fuzzy judgments is rather consistent, and if it is a negative value, it means that the fuzzy judgments are inconsistent. The closer to one, the greater the consistency and compatibility of judgments are. The consistency index is a metric devised to gauge the level of consistency in the provided pairwise comparisons. In essence, λ measures the extent to which the matrix deviates from perfect consistency. While the exact threshold can vary depending on the specific context or the complexity of the decision problem, an index of 0.6 or higher is generally deemed acceptable, implying that the judgments are sufficiently consistent for the decision-making process [49].

**Step A-2.** In this step, the interdependent effects and the global weights of the criteria are calculated. The interdependent effects are asked for by the experts and extracted from the questionnaires. The global weights of the factors are equal to the product of the local weights of the factors and the interdependency matrix.

**Step A-3.** The global weights of the sub-factors are calculated by multiplying the local weight of the sub-factor with the global weights of the relevant factor, calculated in the previous step.

- FANP approach B

The approach presented by [57] for fuzzy prioritization is based on [58,59]’s fuzzy arithmetic means and requires a fuzzy ranking procedure to compare the final fuzzy scores. To determine the final weights of the criteria, the following steps need to be followed:

**Step B-1.** Calculating the fuzzy synthetic extent for the criterion i, denoted as $S_{i}$ (the combined judgment for criterion i relative to all others), as follows [57]:

(7) $${\tilde{S}}_{i}=\left(a_{i},\;b_{i},\;c_{i}\right)=\left(\sum_{j=1}^{n}l_{ij},\;\sum_{j=1}^{n}m_{ij},\;\sum_{j=1}^{n}u_{ij}\right)⨂{\left(\sum_{i=1}^{n}\sum_{j=1}^{n}l_{ij},\;\sum_{i=1}^{n}\sum_{j=1}^{n}m_{ij},\;\sum_{i=1}^{n}\sum_{j=1}^{n}u_{ij}\right)}^{-1},\;\forall i=1,\;\ldots ,\;n$$

**Step B-2.** To obtain criteria weights, the principle of comparison must be considered for fuzzy numbers. The degree of possibility (the likelihood that one fuzzy number is greater than another) of comparing two fuzzy synthetic extents ${\tilde{S}}_{i}\;$ and $\;{\tilde{S}}_{j}$ is defined as follows [57]:

(8) $$V\left({\tilde{S}}_{i}\geq {\tilde{S}}_{j}\right)=1,\;iff\;b_{i}\geq b_{j}$$

(9) $$V\left({\tilde{S}}_{j}\geq {\tilde{S}}_{i}\right)=hgt\left({\tilde{S}}_{i}⋂{\tilde{S}}_{j}\right)=\left\{\begin{array}{c} \frac{a_{i}-c_{j}}{\left(b_{j}-c_{j}\right)-\left(b_{i}-a_{i}\right)},\;a_{i}\leq c_{j}\\ 0\;\;\;\;\;\;\;\;\;\;\;\;\;\;\;\;,\;o.w. \end{array}\right.$$

**Step B-3.** Calculating the relative preference of each criterion (the least possibility that criterion j is worse than any other), denoted as $d_{j}^{\prime }=\underset{i}{\min\;}\left(V\left({\tilde{S}}_{j}\geq {\tilde{S}}_{i}\;\right)\right)$ and then normalizing them to find the criteria weights, $W_{j}=\frac{d_{j}^{\prime }}{\sum_{i=1}^{n}d_{i}^{\prime }\;}.$

**Table 3:** Comparison of FANP approaches A and B.

Aspect	FANP Approach A [49]	FANP Approach B [57]
Methodology	Fuzzy pairwise comparison, transformed into a non-linear program.	Fuzzy synthetic extent and fuzzy ranking procedure.
Step Structure	Three steps: local weight calculation, interdependent effects, and global weight computation.	Three steps: fuzzy synthetic extent, fuzzy ranking, and relative preference calculation.
Mathematical Foundation	Based on fuzzy optimization with constraints.	Based on fuzzy arithmetic means and possibility theory.
Handling of Inconsistency	The consistency index measures deviation from perfect consistency.	Relies on fuzzy ranking to compare final fuzzy scores.
Global Weight Calculation	Local weights are multiplied by the interdependency matrix to obtain global weights.	Final weights are determined by fuzzy synthetic extent and relative preferences.
Complexity	Relatively more computationally intensive due to non-linear programming.	Computationally simpler due to direct use of fuzzy arithmetic.
Advantages	Suitable for complex, interdependent criteria.	Simpler approach, more intuitive ranking of criteria.

## 4. Experimental Results and Discussion

Here, the two approaches of FANP are used to calculate the final weights of the extracted set of factors and sub-factors in Python (versions 3.1 and 3.2). The factors and sub-factors to be used in the model were determined by the expert team. Fuzzy pairwise comparison matrices used to calculate factor and sub-factor weights were also formed by the same team. The application is performed based on the steps provided in the previous section and explained step by step together with the results.

### 4.1. Results of FANP Approach A

**Figure 3:**
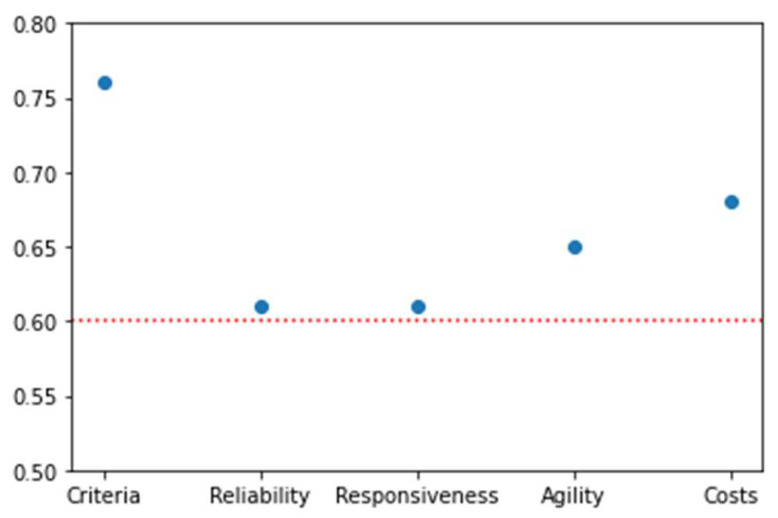
The consistency of the pairwise comparison matrices.

Local and global weights of the factors and sub-factors are calculated using the FANP approach A in this section. The FANP questionnaire was presented to the expert team and was completed by them using the scale provided in Table 2. The aggregated TFNs associated with each comparison are calculated and form the upper triangle of the pairwise comparison matrices. The lower triangle of the pairwise comparison matrices is calculated by inverting the corresponding cells in the upper triangle. For instance, since the aggregated TFN for reliability and agility is calculated (1.31, 1.81, 2.94), the aggregated TFN for agility and reliability is obtained as (0.34, 0.55, 0.76). Obviously, all the TFNs on the main diagonal are (1, 1, 1). In Table 4, the local weights assigned to the primary factors—reliability, responsiveness, agility, and cost—are presented, along with their corresponding consistency index of λ = 0.76. Table 5, Table 6, Table 7 and Table 8 showcase the local weights allocated to the sub-factors linked to the primary factors—reliability, responsiveness, agility, and cost. Figure 3 displays the calculated consistency indices, which determine the consistency of the pairwise comparison matrices. An index of 0.6 or higher is generally deemed acceptable. The next step involves extracting interdependent effects, which are then presented in Table 9.

Using the interdependent weights reported in Table 9, the global weights of the criteria are calculated using the following:

$${GW}_{Criteria}=\left[\begin{array}{c} Reliability\\ Responsiveness\\ Agility\\ Costs \end{array}\right]=\left[\begin{array}{c} \begin{array}{c} \begin{array}{cc} 0.75 & \begin{array}{ccc} 0.09 & 0.11 & 0.05 \end{array} \end{array}\\ \begin{array}{ccc} 0.12 & 0.74 & \begin{array}{cc} 0.15 & 0.01 \end{array} \end{array}\\ \begin{array}{ccc} 0.06 & 0.10 & \begin{array}{cc} 0.66 & 0.11 \end{array} \end{array} \end{array}\\ \begin{array}{ccc} 0.07 & 0.07 & \begin{array}{cc} 0.08 & 0.83 \end{array} \end{array} \end{array}\right]\times \left[\begin{array}{c} 0.34\\ 0.31\\ \begin{array}{c} 0.18\\ 0.17 \end{array} \end{array}\right]=\left[\begin{array}{c} 0.311\\ 0.299\\ 0.189\\ 0.201 \end{array}\right]$$

Global weights for sub-factors are calculated by utilizing interdependent weights of the factors and local weights of the sub-factors. Table 10 shows that the highest score of 0.311 is attributed to reliability, making it the most crucial factor. It is closely followed by responsiveness, which is scored at 0.299. The third and fourth factors, namely costs and agility, are scored at 0.201 and 0.189, respectively. As a result, it can be concluded that the two most important criteria are reliability and responsiveness, while the third and fourth positions are occupied by costs and agility, respectively. In the evaluation of XR systems, the sub-factors that belong to the reliability category are safety, accuracy, user control, and freedom, which are deemed the most important. On the other hand, the sub-factors belonging to the responsiveness category, namely information update gap, interaction method, and feedback, are ranked fourth, fifth, and sixth, respectively. Warranty and depreciation costs are considered the least significant criteria in this matter.

### 4.2. Results of FANP Approach B

In approach B, the criteria weights are determined by utilizing the pairwise comparison matrices from approach A. This is achieved by first calculating the fuzzy synthetic extent for each criterion, as reported in Table 11. Then, the weights for primary factors and sub-factors are derived and are depicted in Table 12, Table 13, Table 14, Table 15 and Table 16. Utilizing these local weights, the global weights are computed and reported in Table 16. According to the findings, reliability ranks as the most significant primary factor, followed by responsiveness, cost, and agility. Within the reliability category, safety, user control and freedom, and accuracy emerge as the top three most crucial sub-factors.

In analyzing the results from Table 10 and Table 17, several key insights emerge. The calculated global weights and ranks for the factors and sub-factors provide a clear picture of the relative importance of each criterion in the selection of XR systems in healthcare.

**Figure 4:**
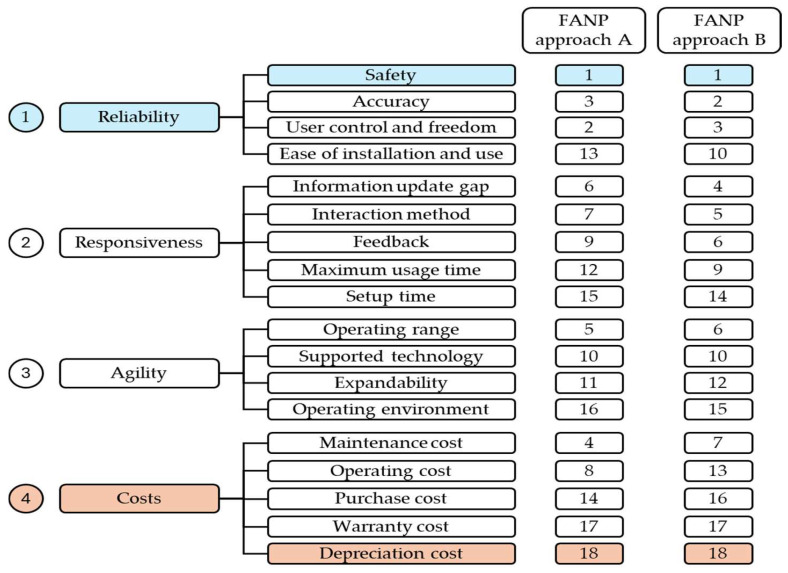
Visual schema comparing sub-criteria rankings across two FANP approaches (A and B) for evaluating XR systems in healthcare. Sub-criteria are grouped under four primary criteria—reliability, responsiveness, agility, and costs—ordered by overall importance (1 = most important).

According to Figure 4, it is evident that ‘Reliability’ is the most significant primary factor, emphasizing the critical nature of system dependability in healthcare applications. Within the reliability category, ‘Safety’ is ranked the highest, underscoring the paramount importance of patient safety in healthcare technology. The high rankings of ‘Accuracy’ and ‘User Control and Freedom’ further highlight the need for precise and user-friendly XR systems.

In the category of ‘Responsiveness’, the ‘Information Update Gap’ and ‘Interaction Method’ are ranked as more critical than other sub-factors like ‘Feedback’ and ‘Setup Time.’ This suggests a preference for up-to-date information and interactive methods in XR systems, which are crucial for real-time applications in healthcare.

For ‘Costs’, ‘Maintenance Cost’ receives a higher weight compared to ‘Operating Cost’ or ‘Purchase Cost’, indicating that long-term maintenance is a more significant concern than initial purchase or operational expenses. This reflects the healthcare industry’s focus on sustainable and cost-effective solutions. The lower significance of ‘Warranty Cost’ and ‘Depreciation Cost’ perhaps points to a lesser concern over the economic lifespan of the technology in favor of performance and maintenance aspects.

Lastly, within the ‘Agility’ category, the primary focus seems to be on ‘Operating Range’, which suggests a preference for XR systems that offer a broad range of operation, essential for diverse healthcare settings.

### 4.3. Comparative Analysis and Managerial Insights

This section presents a comparative analysis of the results obtained from the two state-of-the-art fuzzy approaches of ANP to identify the most critical primary factors and sub-factors for evaluating XR systems in healthcare. FANP approach A offers a distinctive advantage through its innovative use of fuzzy pairwise comparison judgments, replacing exact numerical values with a non-linear programming transformation for the initial fuzzy prioritization problem. Unlike traditional fuzzy prioritization techniques, this approach generates crisp weights by addressing both consistent and inconsistent fuzzy comparison matrices, thereby eliminating the necessity for additional aggregation and ranking procedures. On the other hand, FANP approach B excels in simplicity and ease of implementation. Its straightforward steps make it more accessible compared to other fuzzy approaches. Additionally, this approach accommodates complex interrelationships among decision levels and attributes, allowing for a more comprehensive analysis. However, it relies on fuzzy arithmetic mean and necessitates a fuzzy ranking procedure for comparing final fuzzy scores. Kendall’s tau coefficient and Spearman’s rank correlation coefficient are utilized to evaluate the statistical association between the ranks of the data. These coefficients are computed to examine if there is a notable difference in the rankings of sub-factors. The outcomes of these calculations are presented in Table 18, where the values of Kendall and Spearman coefficients are reported along with their respective *p*-values. Both Kendall’s tau and Spearman’s rank correlation coefficients indicate a strong positive correlation between the sub-factor rankings. The *p*-values for both tests are very small, indicating that the results are statistically significant at conventional levels. The Spearman coefficient is higher than the Kendall coefficient, suggesting that the ranking agreement between the two methods is even stronger when considering the actual rank values rather than just the relative ordering.

**Figure 5:**
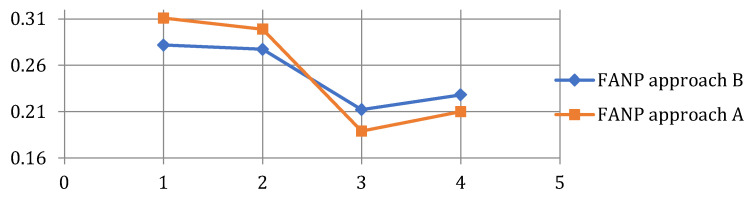
Difference between the primary factors’ local weights.

**Figure 6:**
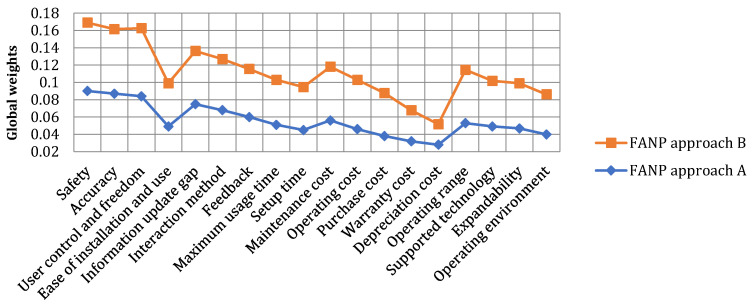
Comparison of global weights between FANP approach A and FANP approach B in the context of XR applications in healthcare. Each point on the line represents the global importance weight of a specific sub-criterion. The orange squares represent results from approach B, and the blue diamonds represent results from approach A. Higher values indicate greater importance in decision-making.

As illustrated in Figure 5, both approaches yield the same ranking of primary factors, highlighting the significance of reliability as the topmost factor, followed by responsiveness, cost, and agility. Figure 6 illustrates that while the top three important sub-factors are identical between the two approaches, their relative order is slightly varied. Both approaches consider safety to be the most critical aspect within the reliability category, but there are discrepancies in the rankings of accuracy and user control and freedom. Both approaches also consider warranty and depreciation costs as the least significant criteria.

The proposed decision model provides a systematic and structured approach to evaluating the performance of XR systems based on the criteria and sub-criteria identified as important for healthcare applications. These criteria include reliability, responsiveness, agility, and cost aspects of these technologies. Using this model, along with the guideline provided in Table 19, healthcare organizations (e.g., hospital management or procurement officers) can make well-informed decisions about selecting the most suitable XR system based on their specific needs and preferences. The model also provides a quantitative basis for comparing different XR systems, enabling decision-makers to prioritize and weigh different factors based on their relative importance. Overall, it can help improve the selection of XR systems in healthcare, leading to better outcomes for patients and providers alike.

## 5. Performance Evaluation of Existing XR Technologies Based on the Proposed Criteria

It is important to note that the relative importance of the evaluation criteria proposed in this study may vary depending on the intended use case of the XR system. For instance, applications in surgical training may prioritize accuracy and feedback responsiveness, while remote collaboration solutions might emphasize operating range and ease of use. Chiang et al. developed a VR simulator aimed at reducing the training time for cardiologists pursuing certification and experience in intracardiac interventions. They assessed the simulator’s efficacy, and their findings were fully consistent with the results of our study. Notably, their evaluation highlighted ‘accuracy’ as the highest priority, followed by ‘responsiveness’ and then ‘cost’ as the next most important factors [60]. However, for the purpose of a consistent and generalizable comparison across various XR technologies, we rely on the weighting derived from the FANP model developed in this study. To assess the performance of widely available XR systems in the market, we selected eight representative devices encompassing a mix of AR, VR, and MR technologies. These include Microsoft HoloLens 2, Meta Quest 3, Magic Leap 2, Varjo XR-4, HTC Vive Pro 2, Apple Vision Pro, Pico 4 Enterprise, and RealWear Navigator 500. Each system was evaluated against the 18 sub-factors categorized under the four primary criteria: reliability, responsiveness, agility, and cost. The scoring for each sub-factor was informed by manufacturer specifications, third-party technical reviews, and relevant literature (see Section 2), with scores normalized on a 1-to-10 scale and then weighted based on the FANP-derived importance. The detailed scoring tables for each XR system are available in Appendix A.

**Figure 7:**
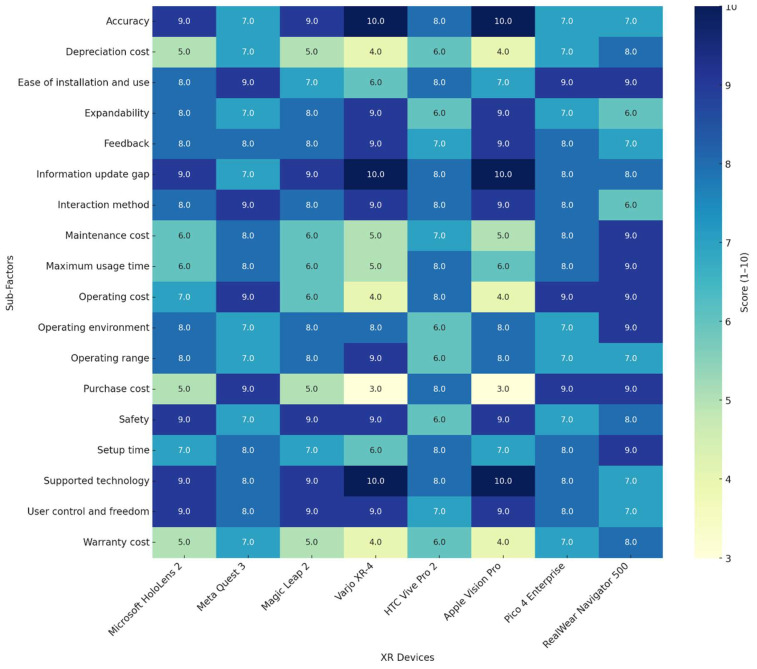
Heatmap displaying expert-assigned scores (on a scale of 1–10) for each sub-criterion across eight XR devices used in healthcare training and simulation. Rows represent 20 sub-criteria, while columns represent different XR devices. Higher scores (shown in darker blue) indicate better performance on the corresponding sub-criteria. The color gradient legend on the right reflects the score scale from 3 (lowest) to 10 (highest).

Figure 7 presents a detailed, at-a-glance comparison of strengths and weaknesses across each criterion and illustrates a trade-off between cost-efficiency and high-end technical capability. Devices such as the Meta Quest 3 and Pico 4 Enterprise offer attractive affordability and ease of use but tend to score slightly lower in clinically critical dimensions like safety and accuracy. In contrast, premium systems such as Apple Vision Pro and Varjo XR-4 deliver top-tier performance but at a significantly higher cost. These distinctions underscore the importance of contextualizing XR system selection based on the operational goals and constraints of the intended healthcare environment.

As displayed in Figure 7, Apple Vision Pro and Microsoft HoloLens 2 consistently demonstrate high scores, with most sub-factor evaluations above 8 out of 10. These systems particularly excel in critical dimensions such as accuracy, information update gap, user control and freedom, and safety. Their uniformly strong performance highlights their suitability for high-stakes healthcare applications, including surgical visualization, complex diagnostics, and real-time guided interventions, where immersive precision and system reliability are paramount. RealWear Navigator 500 and Pico 4 Enterprise, by contrast, show their strengths in cost-related and usability-focused dimensions. These systems exhibit top scores in purchase cost, operating cost, maintenance cost, and ease of installation and use, while still maintaining competitive scores in responsiveness-related sub-factors such as setup time and maximum usage time. Although they do not match the premium devices in terms of spatial fidelity or interaction precision, their affordability and operational durability make them well-suited for deployment in resource-limited healthcare settings, as well as for training, remote collaboration, and telehealth applications.

**Figure 8:**
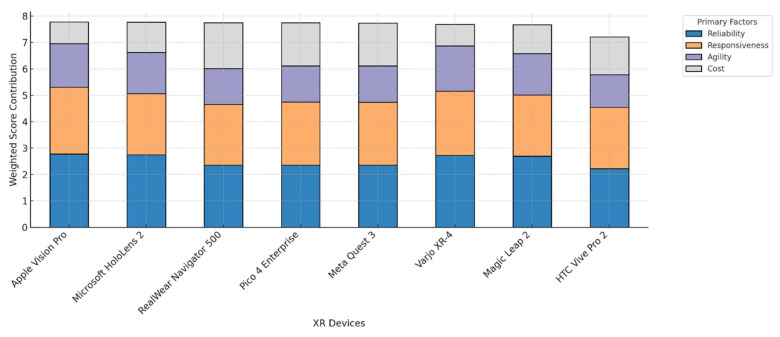
Factors’ contributions to the total score (sorted by the overall score).

As displayed in Figure 7, Varjo XR-4 stands out with a perfect score in both accuracy and supported technology, reflecting its high-resolution display and hybrid MR/VR capabilities. This makes it particularly advantageous in applications requiring visual realism and high-performance computing, such as advanced simulation environments or neurosurgical planning. Similarly, Magic Leap 2 scores highly in areas such as interaction method and safety, aligning it with AR applications like physical therapy, patient education, and in-field visualization. Notably, HTC Vive Pro 2 scores relatively well in usage-related dimensions like ease of installation and maximum usage time but falls behind in several cost and agility-related metrics, including depreciation cost and setup time. This variability suggests that while the device may be suitable for training and demonstration purposes, it may be less optimal for sustained clinical deployment. To better understand how the primary evaluation criteria contribute to the overall performance of each XR system, Figure 8 displays a stacked bar chart summarizing the weighted contributions of reliability, responsiveness, agility, and cost.

Figure 8 reveals not just which XR systems score highest, but how their strengths are structurally composed across the four decision-making criteria. One immediately noticeable insight is that top-performing systems do not rely equally on all factors—rather, each has a unique profile of emphasis. For instance, while Apple Vision Pro and Microsoft HoloLens 2 both sit at the top of the performance ranking, their category distributions show subtle but meaningful differences: Apple Vision Pro demonstrates a relatively more diversified contribution across reliability, responsiveness, and agility, whereas Microsoft HoloLens 2’s strength is more concentrated in responsiveness and reliability. This suggests that Apple’s system may offer greater adaptability across varied environments and use cases, while HoloLens 2 may be more optimal for applications demanding fast, stable interaction in known settings.

**Figure 9:**
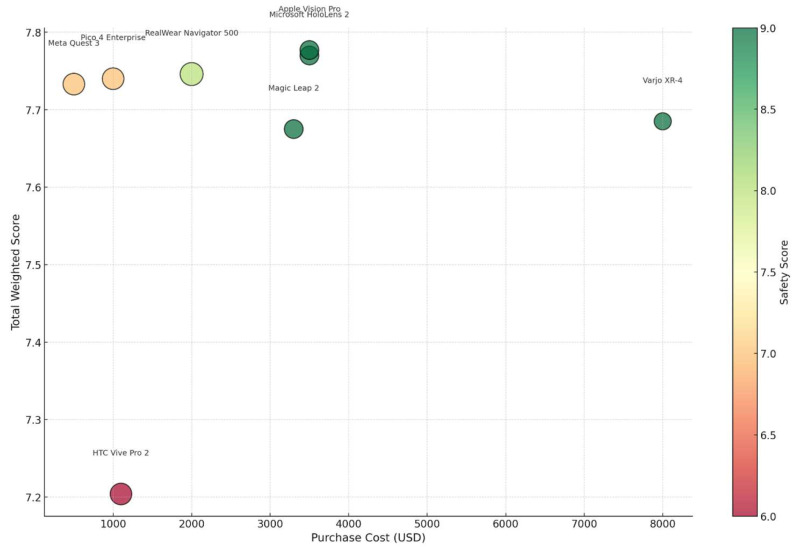
Total score vs. purchase cost: bubble size = max usage time | color = safety score.

Interestingly, the cost contribution is highest among mid-ranking systems such as RealWear Navigator 500 and Pico 4 Enterprise. These systems exhibit a value-driven profile, where high scores are derived in part from low operating and acquisition costs, reinforcing their suitability for scaled implementations, workforce training, or budget-conscious clinical environments. In contrast, high-end systems such as Varjo XR-4 show a minimal cost contribution, reflecting that their performance is achieved through premium technical specifications rather than economic efficiency. This divergence underscores that systems optimized for technical superiority may inherently require more investment, even if their overall performance ranks similarly to more economical alternatives. Another insight emerges from the responsiveness category, which appears as a common strength across nearly all devices—indicating that most XR manufacturers are prioritizing real-time feedback, interaction fluidity, and operational responsiveness. This might be a result of competitive differentiation in user experience, especially as XR expands into healthcare settings where latency, gesture recognition, and feedback are essential for clinician and patient engagement. A complementary view is provided in Figure 9, which visualizes each XR system as a bubble in a 2D plane with purchase cost on the *x*-axis and total weighted score on the *y*-axis.

Figure 9 illustrates a multidimensional lens on XR system performance by simultaneously visualizing purchase cost, total weighted score, safety, and maximum usage time. From this perspective, what becomes clear is that value is not linearly tied to investment—while systems like the Apple Vision Pro and Varjo XR-4 occupy the upper-right region, indicating high performance and high cost, several mid-cost devices such as RealWear Navigator 500 and Pico 4 Enterprise achieve nearly comparable performance scores with significantly lower price points and longer operational durations. Moreover, by encoding safety as color intensity and usage time as bubble size, the plot reveals an important operational insight: some lower-cost devices like RealWear deliver not only economic efficiency but also durability and reliability, key for extended use in resource-constrained or mobile healthcare environments. This shifts the interpretation of “performance” from a purely technical measure to one that incorporates practical usability and sustainability, especially relevant for broader adoption across healthcare contexts. While our evaluation offers a comprehensive comparison of XR devices using a structured set of criteria, several limitations warrant consideration. First, the scoring of individual sub-factors relied on secondary sources such as manufacturer specifications, third-party technical reviews, and peer-reviewed literature. Although these sources are valuable and commonly used in technology assessments, they may not fully capture real-world performance, particularly in clinical settings. To mitigate this limitation, we employed cross-referencing data from multiple independent sources whenever possible to ensure greater accuracy and consistency in the assigned scores. Additionally, all scores were normalized to a consistent 1–10 scale to allow meaningful comparison across devices and sub-criteria. While we aimed to standardize the scoring process, some subjectivity remains unavoidable. To reduce this risk, the weighting of sub-factors was determined using the FANP, which incorporates expert judgment in a structured and consistent manner. This approach ensures that the influence of any individual subjective assessment is moderated by collective expert input.

## 6. Conclusions

Although the capabilities of XR systems in healthcare have not yet been fully explored, they can offer significant benefits for improving patient care in innovative and effective ways. We propose the first comprehensive FANP-based decision support system (DSS) specifically designed to evaluate XR technologies in healthcare. This framework integrates the SCOR model with FANP to systematically assess XR technologies by prioritizing key evaluation factors across four dimensions—reliability, responsiveness, agility, and cost—encompassing a total of 18 criteria. These factors are identified by a hybridization of the SCOR model, a literature survey, and experts’ knowledge and experience. To determine the importance of these factors and sub-factors, an integrated fuzzy ANP approach is used. Two fuzzy approaches for the ANP method from the literature are utilized, and the results are analyzed to compare the relative weights of all the identified factors. After calculating Kendall’s Tau and Spearman’s rank correlation coefficients, the results indicate a robust positive correlation between the rankings obtained by the two fuzzy approaches. The application of this framework to eight prominent XR systems highlighted performance trade-offs and helped identify domain-specific system strengths. Insights from the comparative analysis and sensor-based evaluation were discussed to guide procurement and development decisions in healthcare contexts. By utilizing the proposed Decision Support System, the selection of XR systems in healthcare can be enhanced, resulting in improved outcomes for both patients and providers. While this research provides a solid foundation for XR system evaluation in healthcare, there are natural areas for extension. The expert-driven scoring and weighting process, while rigorous, could be further strengthened by incorporating a broader range of perspectives or real-time user feedback. Additionally, as this study focused on a structured pre-market evaluation, future work could complement the model with longitudinal benchmarking using field data such as clinician satisfaction, patient outcomes, and user analytics. Tailoring the framework for specific clinical applications and integrating evolving sensing and AI technologies also represent meaningful directions to enrich and adapt the model for ongoing advancements in XR.

## Figures and Tables

**Table 1:** Sensors and sensing technologies in XR systems and their impact on performance.

Sensor Type	Functionality	Used In	Impact on Performance	Healthcare Application Relevance
IMU	Tracks motion (acceleration, rotation)	VR, AR, MR	Essential for motion tracking and head/body orientation	Used in surgical simulation and rehabilitation
Optical Tracking Cameras	Detects external markers or body movement	VR, MR	Enables accurate spatial positioning of users and objects	Crucial in VR-based procedural training and motion analysis [7]
Depth Sensors (e.g., ToF, RGB-D)	Measures distance to objects in real-time	AR, MR	Enhances spatial mapping and occlusion handling	Key for image-guided surgery and spatial visualization
LiDAR	High-precision environmental mapping	AR, MR	Improves SLAM, depth perception, and scene understanding	Beneficial in remote diagnostics and navigation [13]
Eye Tracking	Tracks gaze direction and eye focus	MR, high-end VR	Enables attention-aware interfaces and foveated rendering	Supports cognitive diagnostics and user attention monitoring [5]
Hand Tracking/Gesture Sensors	Detects hand position and gestures	MR, AR, some VR	Allows natural user interaction without physical controllers	Enables sterile interaction in surgical planning and teleconsultation [8]
Physiological Sensors	Measures vitals (e.g., heart rate, GSR, EEG)	MR, research VR	Adds biometric feedback for adaptive environments	Applied in mental health therapy, stress monitoring, and pain distraction [9,31]
SLAM (Sensor Fusion Method)	Maps and localizes the user in an environment	AR, MR	Foundation for anchoring virtual content to real-world space	Essential for context-aware patient visualization and in-field diagnostics [13]

**Table 2:** Linguistic scales for importance [51].

Linguistic Importance Scale	TFN Scale	TFN Reciprocal Scale
Equally important	(1, 1, 1)	(1, 1, 1)
Weakly more important	(1, 3/2, 2)	(1/2, 2/3, 1)
Moderately more important	(3/2, 2, 5/2)	(2/5, 1/2, 2/3)
Strongly more important	(2, 5/2, 3)	(1/3, 2/5, 1/2)
Extremely more important	(5/2, 3, 7/2)	(2/7, 1/3, 2/5)

**Table 4:** Local weights of primary factors under FANP Approach A.

Criteria	Reliability	Responsiveness	Agility	Cost	Local Weight
Reliability	(1, 1, 1)	(0.6, 1.07, 5.26)	(1.31, 1.81, 2.94)	(0.64, 1.15, 5.26)	0.34
Responsiveness	(0.19, 0.93, 1.67)	(1, 1, 1)	(1.37, 1.75, 2.44)	(0.68, 1.16, 3.84)	0.31
Agility	(0.34, 0.55, 0.76)	(0.41, 0.57, 0.73)	(1, 1, 1)	(0.78, 1.16, 2.27)	0.18
Cost	(0.19, 0.87, 1.55)	(0.26, 0.86, 1.46)	(0.44, 0.86, 1.28)	(1, 1, 1)	0.17

**Table 5:** Local weights of sub-factor reliability under FANP Approach A.

Reliability	Accuracy	Safety	Ease of Installation and Use	User Control and Freedom	Local Weight
Accuracy	(1, 1, 1)	(0.41, 0.69, 2.17)	(0.99, 1.61, 4.34)	(0.53, 0.91, 3.22)	0.28
Safety	(0.46, 1.45, 2.44)	(1, 1, 1)	(1, 1.44, 2.63)	(0.85, 1.23, 2.22)	0.29
Ease of installation and use	(0.23, 0.62, 1.01)	(0.38, 0.69, 1)	(1, 1, 1)	(0.32, 0.46, 0.84)	0.16
User control and freedom	(0.31, 1.09, 1.87)	(0.45, 0.81, 1.17)	(1.18, 2.15, 3.12)	(1, 1, 1)	0.27

**Table 6:** Local weights of sub-factor responsiveness under FANP Approach A.

Responsiveness	Feedback	Setup Time	Maximum Usage Time	Interaction Method	Information Update Gap	Local Weight
Feedback	(1, 1, 1)	(0.95, 1.51, 3.70)	(0.74, 1.08, 2.04)	(0.41, 0.66, 1.66)	(0.47, 0.66, 1.15)	0.20
Setup time	(0.27, 0.66, 1.05)	(1, 1, 1)	(0.45, 0.73, 1.96)	(0.48, 0.81, 2.5)	(0.38, 0.52, 0.83)	0.15
Maximum usage time	(0.49, 0.92, 1.35)	(0.51, 1.37, 2.23)	(1, 1, 1)	(0.45, 0.63, 1.19)	(0.44, 0.66, 1.3)	0.17
Interaction method	(0.6, 1.5, 2.4)	(0.4, 1.23, 2.06)	(0.84, 1.53, 2.22)	(1, 1, 1)	(0.69, 0.96, 1.56)	0.23
Information update gap	(0.87, 1.5, 2.13)	(1.2, 1.92, 2.64)	(0.77, 1.51, 2.25)	(0.64, 1.04, 1.44)	(1, 1, 1)	0.25

**Table 7:** Local weights of sub-factor agility under FANP Approach A.

Agility	Supported Tech.	Expandability	Operating Environment	Operating Range	Local Weight
Supported technology	(1, 1, 1)	(0.64, 0.9, 1.51)	(1.02, 1.37, 2.08)	(0.43, 0.66, 1.51)	0.26
Expandability	(0.66, 1.11, 1.56)	(1, 1, 1)	(0.69, 1, 1.81)	(0.64, 0.84, 1.21)	0.25
Operating environment	(0.48, 0.73, 0.98)	(0.55, 1, 1.45)	(1, 1, 1)	(0.62, 0.83, 1.25)	0.21
Operating range	(0.66, 1.5, 2.34)	(0.82, 1.19, 1.56)	(0.8, 1.2, 1.6)	(1, 1, 1)	0.28

**Table 8:** Local weights of sub-factor cost under FANP Approach A.

Costs	Purchase Cost	Operating Cost	Maintenance Cost	Warranty Cost	Depreciation Cost	Local Weight
Purchase	(1, 1, 1)	(0.7, 0.91, 1.42)	(0.45, 0.75, 1.31)	(0.73, 1.1, 1.66)	(1.13, 1.51, 2.07)	0.19
Operating	(0.7, 1.1, 1.42)	(1, 1, 1)	(0.56, 0.95, 1.55)	(1.14, 1.61, 2.13)	(1.18, 1.67, 2.27)	0.23
Maintenance	(0.76, 1.33, 2.22)	(0.64, 1.05, 1.78)	(1, 1, 1)	(1.4, 1.68, 2.43)	(1.69, 2.08, 2.70)	0.28
Warranty	(0.6, 0.91, 1.36)	(0.47, 0.62, 0.87)	(0.41, 0.59, 0.71)	(1, 1, 1)	(0.91, 1.15, 1.42)	0.16
Depreciation	(0.42, 0.62, 0.88)	(0.44, 0.6, 0.84)	(0.37, 0.48, 0.59)	(0.7, 0.87, 1.09)	(1, 1, 1)	0.14

**Table 9:** The interdependent weights of the criteria.

Criteria	Reliability	Responsiveness	Agility	Costs
Reliability	0.79	0.09	0.11	0.05
Responsiveness	0.08	0.74	0.15	0.01
Agility	0.06	0.10	0.66	0.11
Costs	0.07	0.07	0.08	0.82

**Table 10:** Global weight and rank for factors and sub-factors under FANP Approach A.

Criteria	Sub-Factors	Global Weight	Rank
Reliability	Safety	0.090	1
	Accuracy	0.087	2
	User control and freedom	0.084	3
	Ease of installation and use	0.049	10
Responsiveness	Information update gap	0.075	4
	Interaction method	0.068	5
	Feedback	0.060	6
	Maximum usage time	0.051	9
	Setup time	0.045	14
Costs	Maintenance cost	0.056	7
	Operating cost	0.046	13
	Purchase cost	0.038	16
	Warranty cost	0.032	17
	Depreciation cost	0.028	18
Agility	Operating range	0.053	8
	Supported technology	0.049	10
	Expandability	0.047	12
	Operating environment	0.040	15

**Table 11:** The fuzzy synthetic extent for primary factors and sub-factors.

Primary Factors	Sub-Factors	Fuzzy Synthetic Extent
Reliability		(0.11, 0.30, 1.29)
	Accuracy	(0.10, 0.25, 0.97)
	Safety	(0.11, 0.30, 0.75)
	Ease of installation	(0.06, 0.16, 0.35)
	User control and freedom	(0.10, 0.29, 0.64)
Responsiveness		(0.10, 0.29, 0.80)
	Information update gap	(0.11, 0.16, 0.55)
	Interaction method	(0.08, 0.15, 0.54)
	Feedback	(0.08, 0.12, 0.56)
	Maximum usage time	(0.07, 0.11, 0.41)
	Setup time	(0.06, 0.09, 0.43)
Costs		(0.06, 0.21, 0.47)
	Maintenance cost	(0.15, 0.20, 0.50)
	Operating cost	(0.13, 0.18, 0.41)
	Purchase cost	(0.11, 0.15, 0.37)
	Warranty cost	(0.09, 0.12, 0.26)
	Depreciation cost	(0.08, 0.10, 0.22)
Agility		(0.08, 0.20, 0.42)
	Operating range	(0.14, 0.30, 0.54)
	Supported technology	(0.14, 0.24, 0.51)
	Expandability	(0.13, 0.24, 0.46)
	Operating environment	(0.12, 0.22, 0.39)

**Table 12:** Local weights of primary factors under FANP Approach B.

Criteria	Comparing Synthetic Index				Raw Weights	Local Weight
	Reliability	Responsiveness	Agility	Cost		
Reliability	-	1.00	1.00	1.00	1.00	0.282
Responsiveness	0.98	-	1.00	1.00	0.98	0.277
Agility	0.75	0.78	-	0.95	0.75	0.212
Cost	0.81	0.83	1.00	-	0.81	0.228

**Table 13:** Local and raw weights of sub-factor reliability under FANP Approach B.

Reliability	Comparing Synthetic Index				Raw Weights	Local Weight
	Accuracy	Safety	Ease of Installation and Use	User Control and Freedom		
Accuracy	-	0.94	1.00	0.95	0.94	0.26
Safety	1.00	-	1.00	1.00	1.00	0.28
Ease of installation and use	0.75	0.63	-	0.65	0.63	0.18
User control and freedom	1.00	0.99	1.00	-	0.99	0.28

**Table 14:** Local and raw weights of sub-factor responsiveness under FANP Approach B.

Responsiveness	Comparing Synthetic Index					Raw Weights	Local Weight
	Feedback	Setup Time	Maximum Usage Time	Interaction Method	Information Update Gap		
Feedback	-	1.00	1.00	0.94	0.90	0.90	0.20
Setup time	0.93	-	0.95	0.86	0.81	0.81	0.18
Maximum usage time	0.98	1.00	-	0.90	0.85	0.85	0.19
Interaction method	1.00	1.00	1.00	-	0.96	0.96	0.21
Information update gap	1.00	1.00	1.00	1.00	-	1.00	0.22

**Table 15:** Local and raw weights of sub-factor agility under FANP Approach B.

Agility	Comparing Synthetic Index				Raw Weights	Local Weight
	Operating Range	Operating Range	Operating Range	Operating Range		
Supported technology	-	1.00	1.00	0.86	0.86	0.25
Expandability	1.00	-	1.00	0.85	0.85	0.25
Operating environment	0.92	0.92	-	0.75	0.75	0.22
Operating range	1.00	1.00	1.00	-	1.00	0.29

**Table 16:** Local and raw weights of sub-factor cost under FANP Approach B.

Costs	Comparing Synthetic Index					Raw Weights	Local Weight
	Purchase Cost	Operating Cost	Maintenance Cost	Warranty Cost	Depreciation Cost		
Purchase cost	-	0.89	0.80	1.00	1.00	0.80	0.22
Operating cost	1.00	-	0.92	1.00	1.00	0.92	0.25
Maintenance cost	1.00	1.00	-	1.00	1.00	1.00	0.27
Warranty cost	0.84	0.70	0.58	-	1.00	0.58	0.16
Depreciation cost	0.68	0.53	0.38	0.86	-	0.38	0.10

**Table 17:** Global weight and rank for factors and sub-factors under FANP Approach B.

Criteria	Sub-Factors	Global Weight	Rank
Reliability	Safety	0.079	1
	Accuracy	0.074	3
	User control and freedom	0.078	2
	Ease of installation and use	0.050	13
Responsiveness	Information update gap	0.061	6
	Interaction method	0.059	7
	Feedback	0.055	9
	Maximum usage time	0.052	12
	Setup time	0.050	15
Costs	Maintenance cost	0.062	4
	Operating cost	0.057	8
	Purchase cost	0.050	14
	Warranty cost	0.036	17
	Depreciation cost	0.024	18
Agility	Operating range	0.079	5
	Supported technology	0.074	10
	Expandability	0.078	11
	Operating environment	0.050	16

**Table 18:** Result of statistical testing.

Test	Correlation Coefficients	*p*-Value
Kendall’s Tau	0.773	7.72 × 10^−6^
Spearman’s	0.912	1.28 × 10^−7^

**Table 19:** General Guideline for using the XR system evaluation model.

1- Understand the Decision Context: - Consider the Intended Use Case: The importance of different evaluation criteria (reliability, responsiveness, agility, and cost) may vary depending on the specific application of the XR system. For example, reliability may be critical in healthcare applications, while cost may be a larger factor in industries with tighter budgets. - Weights Based on Needs: The model uses FANP-derived weights to provide a standardized comparison. 2- Select XR Systems to Evaluate: - Identify Relevant Technologies: The model has been applied to a representative set of XR systems from various manufacturers, including AR, VR, and MR technologies. - Ensure Comprehensive Coverage: Ensure that the selected systems represent a broad range of XR capabilities relevant to the intended application. 3- Evaluate Systems Using Established Criteria: - Criteria for Evaluation: Each system is assessed across four primary criteria: reliability, responsiveness, agility, and cost, with 18 sub-factors under these categories. - Rate Systems Based on Available Data: Manufacturer specifications, third-party reviews, and technical literature are used to rate each system on a scale of 1 to 10 for each sub-factor. - Normalize and Weight Scores: Scores are normalized on a 1-to-10 scale and then weighted based on the FANP-derived importance of each criterion, ensuring consistency and allowing for a fair comparison. 4- Compare and Analyze the Results: - Quantitative Comparison: Weighted scores are used to compare different XR systems. The model provides a clear, data-driven approach to highlight the strengths and weaknesses of each system. - Identify Best Fit: Based on the evaluation results, prioritize systems that best meet the intended needs, ensuring alignment with operational goals and budget constraints.

## Data Availability

Raw data are unavailable due to privacy or ethical restrictions. Deidentified data can be made available upon request.

## Abbreviations

The following abbreviations are used in this manuscript:

XR	Extended Reality
SCOR	Supply Chain Operations Reference
ANP	Analytic Network Process
AR	Augmented Reality
VR	Virtual Reality
MR	Mixed Reality
PTSD	Post-Traumatic Stress Disorder
ASD	Autism Spectrum Disorder
IMU	Inertial Measurement Units
LiDAR	Light Detection and Ranging
RGB-D	Red, green, blue, and depth
SLAM	Simultaneous Localization and Mapping
DSS	Decision Support System
FANP	Fuzzy Analytical Network Process
AI	Artificial Intelligence
TOF	Time of Flight
EEG	Electroencephalography
GSR	Galvanic Skin Response
TFN	Triangular Fuzzy Number
FDA	Food and Drug Administration
PC	Personal Computer
SDK	Software Development Kit
OLED	Organic Light-Emitting Diode
API	Application Programming Interface
DoF	Degree of Freedom

## Appendix A

**Table A1:** HoloLens II.

	Score	Justification
Accuracy	9	Clinically used; FDA clearance for some applications.
Depreciation cost	5	Excellent spatial mapping and tracking accuracy.
Ease of installation and use	8	Hand/voice control and gaze tracking.
Expandability	8	Self-contained and guided setup; minimal cabling.
Feedback	8	Integrates with Azure, Teams, and cloud platforms.
Information update gap	9	Hand gestures, voice, and eye tracking.
Interaction method	8	Clear visual and audio feedback, haptics limited.
Maintenance cost	6	~2–3 h per charge.
Maximum usage time	6	Moderate setup; user-friendly interface.
Operating cost	7	Designed for large indoor spaces.
Operating environment	8	Strong MR capabilities.
Operating range	8	Enterprise API and developer tools available.
Purchase cost	5	Built for hospitals, clinics, and industry.
Safety	9	Relatively higher for enterprise support.
Setup time	7	Some subscription models depend on apps.
Supported technology	9	~$3500 retail price.
User control and freedom	9	Enterprise support available.
Warranty cost	5	High upfront cost, but durable.

**Table A2:** Meta Quest 3.

	Score	Justification
Accuracy	7	Comfortable and safe for general use; limited clinical testing.
Depreciation cost	7	Decent for gaming/training; lacks professional-grade sensors.
Ease of installation and use	9	Full hand tracking and controller options.
Expandability	7	Plug-and-play; very user-friendly.
Feedback	8	Consumer cloud integration (Meta/Quest account).
Information update gap	7	Hand tracking, controllers, and voice.
Interaction method	9	Haptics and immersive visuals/audio.
Maintenance cost	8	2.5–3 h typical.
Maximum usage time	8	Rapid onboarding.
Operating cost	9	Large indoor spaces.
Operating environment	7	Excellent VR; solid passthrough MR.
Operating range	7	Strong dev ecosystem via Meta SDKs.
Purchase cost	9	Good for training, limited for clinical use.
Safety	7	Low upkeep; robust hardware.
Setup time	8	No enterprise subscription required.
Supported technology	8	~$500–600.
User control and freedom	8	Basic consumer coverage.
Warranty cost	7	High resale market; moderate depreciation.

**Table A3:** Magic Leap 2.

	Score	Justification
Accuracy	9	Designed with FDA-clearable medical apps in mind.
Depreciation cost	5	Great spatial awareness, depth sensing.
Ease of installation and use	7	Eye tracking, hand gestures, controller.
Expandability	8	Requires tethered compute pack; steeper learning curve.
Feedback	8	Real-time data flow, fast processing.
Information update gap	9	Eye tracking, controller, hand gestures.
Interaction method	8	Excellent visuals and audio, limited haptics.
Maintenance cost	6	~3.5 h; tethered, so not mobile-friendly.
Maximum usage time	6	Modular setup; takes longer than standalone.
Operating cost	6	Strong indoors; light-controlled environments.
Operating environment	8	Advanced AR/MR capabilities.
Operating range	8	SDKs and enterprise support are available.
Purchase cost	5	Designed for med/industrial/enterprise use.
Safety	9	Tethered system = more upkeep.
Setup time	7	Licenses for dev tools; enterprise services.
Supported technology	9	~$3300+.
User control and freedom	9	Enterprise support available.
Warranty cost	5	High initial cost and niche resale.

**Table A4:** Varjo XR-4.

	Score	Justification
Accuracy	10	Designed for simulation and clinical settings.
Depreciation cost	4	Industry-best visual fidelity; true-to-life imaging.
Ease of installation and use	6	Accurate tracking but tethered.
Expandability	9	PC-tethered setup, requires base stations.
Feedback	9	Lightning-fast data rendering.
Information update gap	10	Eye tracking, controllers, and hand tracking.
Interaction method	9	Visual/audio fidelity unmatched.
Maintenance cost	5	Tethered to PC; dependent on external power.
Maximum usage time	5	Requires external sensors and a PC.
Operating cost	4	Excellent in large, defined spaces.
Operating environment	8	True VR + full pass-through MR.
Operating range	9	Advanced SDKs; open dev tools.
Purchase cost	3	Built for simulators and labs.
Safety	9	Complex systems = higher upkeep.
Setup time	6	Requires a high-end PC and support tools.
Supported technology	10	~$6000–10,000+.
User control and freedom	9	Professional plans are available but expensive.
Warranty cost	4	Niche resale market.

**Table A5:** HTC Vive Pro-2.

	Score	Justification
Accuracy	8	Generally safe, but the physical setup is demanding.
Depreciation cost	6	Excellent tracking with base stations.
Ease of installation and use	8	Good control; lacks portability.
Expandability	6	Requires base stations but is well-documented.
Feedback	7	Low latency when set up properly.
Information update gap	8	Controllers, SteamVR compatible.
Interaction method	8	Haptics and good visual/audio quality.
Maintenance cost	7	PC-powered; session-limited only by comfort.
Maximum usage time	8	Time-intensive, but smooth once done.
Operating cost	8	Depends on base station setup.
Operating environment	6	VR only.
Operating range	6	Dev tools exist, but the ecosystem is smaller.
Purchase cost	8	Indoor use, some medical training.
Safety	6	Durable hardware.
Setup time	8	No additional subscriptions needed.
Supported technology	8	~$1000–1200 range.
User control and freedom	7	Basic consumer-level support.
Warranty cost	6	Moderate resale value.

**Table A6:** Apple Vision Pro.

	Score	Justification
Accuracy	10	Designed with Apple’s safety-first ecosystem, with advanced sensors and privacy-centric architecture.
Depreciation cost	4	Industry-leading dual 4K micro-OLED displays with eye tracking and depth precision.
Ease of installation and use	7	Excellent gesture/eye tracking and fluid control model.
Expandability	9	High setup complexity, but guided setup helps mitigate.
Feedback	9	Seamless iCloud and device sync, ultra-low latency.
Information update gap	10	Eye tracking, hand gestures, and voice via Siri—top-tier.
Interaction method	9	Multi-sensory (visual, spatial audio, haptic) response system.
Maintenance cost	5	~2 h on battery; longer with external pack.
Maximum usage time	6	Requires calibration but is streamlined via software.
Operating cost	4	Indoor primary use, excellent for open space environments.
Operating environment	8	Supports AR and VR natively with realityOS.
Operating range	8	Strong Apple ecosystem integration + API support.
Purchase cost	3	Designed for controlled spaces but robust in various light.
Safety	9	AppleCare+ is available, but the cost is high.
Setup time	7	Accessories, software licensing, and the Apple ecosystem are required.
Supported technology	10	Extent of AR, VR, MR, or hybrid capabilities
User control and freedom	9	Navigation flexibility, undo options, intuitive control
Warranty cost	4	Extended warranty availability and pricing

**Table A7:** Pico 4.

	Score	Justification
Accuracy	7	Comfortable build, good privacy policies.
Depreciation cost	7	Solid tracking, not as precise as Varjo or HoloLens.
Ease of installation and use	9	6DoF movement and controller input.
Expandability	7	Easy setup, no base stations needed.
Feedback	8	Good enterprise networking features.
Information update gap	8	Controllers and limited hand tracking.
Interaction method	8	Visual + audio feedback; tactile response limited.
Maintenance cost	8	~2.5–3 h.
Maximum usage time	8	Fast onboarding.
Operating cost	9	Large-scale indoor spaces.
Operating environment	7	VR; AR through pass-through only.
Operating range	7	Enterprise SDK, but smaller community.
Purchase cost	9	Office, clinical, training.
Safety	7	Generally durable, low maintenance.
Setup time	8	No platform lock-in.
Supported technology	8	~$900–1200.
User control and freedom	8	Enterprise support available.
Warranty cost	7	Moderate depreciation rate.

**Table A8:** RealWear Navigator 500.

	Score	Justification
Accuracy	7	Ruggedized and designed for high-risk industrial/medical settings.
Depreciation cost	8	Less spatial accuracy due to monocular design.
Ease of installation and use	9	Voice-only control—limits complexity but adds safety.
Expandability	6	Wearable, quick start.
Feedback	7	Cloud connectivity optimized for real-time collaboration.
Information update gap	8	Voice-only. No hand or eye-tracking.
Interaction method	6	Limited to visual/audio output.
Maintenance cost	9	Swappable batteries allow extended use.
Maximum usage time	9	Plug-and-go for most users.
Operating cost	9	Indoor and semi-outdoor use.
Operating environment	9	Primarily AR; limited MR/VR functions.
Operating range	7	Supports Android-based apps; SDK is limited.
Purchase cost	9	Extreme environments, including sterile/hazardous zones.
Safety	8	Rugged design = low failure rate.
Setup time	9	No high-cost accessories needed.
Supported technology	7	~$2000—cost-effective.
User control and freedom	7	Enterprise extended support available.
Warranty cost	8	Low depreciation: hardware stays useful longer.

## Appendix B. Questionnaire

Dear Sir/Madam,

This questionnaire is a part of a research project titled “A multi-criteria decision-making framework for extended reality (XR) systems’ selection in healthcare”. If you choose to take part in this study, you will be asked to rank a set of criteria for evaluating extended reality systems according to a hierarchical model as well as to evaluate the effect(s) of those criteria on each other. You have the option of not answering some of the questions and remaining in the study, and you can withdraw your participation at any time. It takes a maximum of 15 min to complete the questionnaire.

Thank you.

Our model consists of four criteria (i.e., reliability, responsiveness, agility, and costs) and 18 sub-criteria, defined as follows:

**Reliability**: The ability to perform tasks as expected.

1. Accuracy: the degree to which the result of a measurement or calculation conforms to the correct value or a standard. This definition includes concepts such as precision and recall.
2. Safety: the condition or state of being free from harm, injury, or loss.
3. Ease of installation and use: this includes features such as weight, if one or both hands are free during usage, and help and documentation.
4. User control and freedom: the ability of the user to freely move around and perform intended tasks with full control.

**Responsiveness:** The speed and timeliness of service delivery.

1. Feedback: refers to the response time of the system and its ability to provide real-time feedback.
2. Setup time: the time interval necessary to prepare the system for operation.
3. Max usage time: the maximum time that the system can operate without interruption.
4. Interaction method: outlines the usability of the specific XR system. It can be vocal, gestural, touching, physiological, or a combination of more than one method.
5. Information update gap: how fast the XR system allows updating information from/to the central system, thus supporting a more responsive process.

**Agility:** The capacity to effectively sense and respond to external influences/stimuli.

1. Supported tech: it can support augmented reality, virtual reality, mixed reality, or a combination of all.
2. Expandability: the degree to which a system could easily extend or upgrade with new functionalities and abilities to address new requirements.
3. Operating environment: refers to the environments (indoor, outdoor, and severe) in which the system can operate.
4. Operating range: the maximum allowable area where the system can operate.

**Costs:** All costs associated with operating the processes.

1. Purchase cost: the total costs paid for the acquisition of the XR system.
2. Operating cost: all the required costs related to the operation of the XR system.
3. Maintenance cost: the total costs of preserving the system.
4. Warranty cost: the cost paid for obtaining a warranty for the XR system.
5. Depreciation cost: the reduction in the initial value of the machinery resulting from its exploitation.

### Appendix B1. Part One: Compare the Importance

In this section, you are expected to compare the importance of the criteria and the criterion. For instance, if you believe that in an XR system reliability is **extremely more important** than responsiveness, you can fill out number 5 on the left side.

Criteria	5	4	3	2	1	2	3	4	5	Criteria
Reliability										Responsiveness

Also, if you believe reliability and agility are equally important, you can choose 1.

Criteria	5	4	3	2	1	2	3	4	5	Criteria
Reliability										Agility

You can rank all other criteria and sub-criteria similarly.

**COMPARE CRITERIA**

Criteria	5	4	3	2	1	2	3	4	5	Criteria
Reliability										Responsiveness
Reliability										Agility
Reliability										Costs
Responsiveness										Agility
Responsiveness										Costs
Agility										Costs

**COMPARE SUB-CRITERIA 1 (Reliability)**

Sub-Criteria	5	4	3	2	1	2	3	4	5	Sub-Criteria
Accuracy										Safety
Accuracy										Ease of installation
Accuracy										User control
Safety										Ease of installation
Safety										User control
Ease of installation										User control

**COMPARE SUB-CRITERIA 2 (Responsiveness)**

Sub-Criteria	5	4	3	2	1	2	3	4	5	Sub-Criteria
Feedback										Setup time
Feedback										Max usage time
Feedback										Interaction method
Feedback										Information update gap
Setup time										Max usage time
Setup time										Interaction method
Setup time										Information update gap
Max usage time										Interaction method
Max usage time										Information update gap
Interaction method										Information update gap

**COMPARE SUB-CRITERIA 3 (Agility)**

Sub-Criteria	5	4	3	2	1	2	3	4	5	Sub-Criteria
Supported tech										Expandability
Supported tech										Operating environment
Supported tech										Operating range
Expandability										Operating environment
Expandability										Operating range
Operating environment										Operating range

**COMPARE SUB-CRITERIA 4 (Costs)**

Sub-Criteria	5	4	3	2	1	2	3	4	5	Sub-Criteria
Purchase cost										Operating cost
Purchase cost										Maintenance cost
Purchase cost										Warranty cost
Purchase cost										Depreciation cost
Operating cost										Maintenance cost
Operating cost										Warranty cost
Operating cost										Depreciation cost
Maintenance cost										Warranty cost
Maintenance cost										Depreciation cost
Warranty cost										Depreciation cost

### Appendix B2. Part Two: Evaluating the Effect of the Criteria on Each Other

In this section, you are expected to evaluate the effect(s) of the criteria on each other.

**Criteria**	**No Effect**	**Little Effect**	**Some Effect**	**High Effect**	**Extreme Effect**
Reliability on Responsiveness					
Responsiveness on Reliability					
Reliability on Agility					
Agility on Reliability					
Reliability on Costs					
Costs on Reliability					
Responsiveness on Agility					
Agility on Responsiveness					
Responsiveness on Costs					
Costs on Responsiveness					
Agility on Costs					
Costs on Agility					

**Additional Comments**
